# Genomic and Phenotypic Comparisons Reveal Distinct Variants of *Wolbachia* Strain *w*AlbB

**DOI:** 10.1128/aem.01412-22

**Published:** 2022-11-01

**Authors:** Julien Martinez, Perran A. Ross, Xinyue Gu, Thomas H. Ant, Shivan M. Murdochy, Lily Tong, Ana da Silva Filipe, Ary A. Hoffmann, Steven P. Sinkins

**Affiliations:** a MRC-University of Glasgow Centre for Virus Research, Glasgow, United Kingdom; b Pest and Environmental Adaptation Research Group, Bio21 Institute, the University of Melbourne, Parkville, VIC, Australia; University of Nebraska—Lincoln

**Keywords:** *Aedes*, comparative genomics, *Wolbachia*, arbovirus control

## Abstract

The intracellular bacterium *Wolbachia* inhibits virus replication and is being harnessed around the world to fight mosquito-borne diseases through releases of mosquitoes carrying the symbiont. *Wolbachia* strains vary in their ability to invade mosquito populations and suppress viruses in part due to differences in their density within the insect and associated fitness costs. Using whole-genome sequencing, we demonstrate the existence of two variants in *w*AlbB, a *Wolbachia* strain being released in natural populations of Aedes aegypti mosquitoes. The two variants display striking differences in genome architecture and gene content. Differences in the presence/absence of 52 genes between variants include genes located in prophage regions and others potentially involved in controlling the symbiont’s density. Importantly, we show that these genetic differences correlate with variation in *w*AlbB density and its tolerance to heat stress, suggesting that different *w*AlbB variants may be better suited for field deployment depending on local environmental conditions. Finally, we found that the *w*AlbB genome remained stable following its introduction in a Malaysian mosquito population. Our results highlight the need for further genomic and phenotypic characterization of *Wolbachia* strains in order to inform ongoing *Wolbachia*-based programs and improve the selection of optimal strains in future field interventions.

**IMPORTANCE** Dengue is a viral disease transmitted by *Aedes* mosquitoes that threatens around half of the world population. Recent advances in dengue control involve the introduction of *Wolbachia* bacterial symbionts with antiviral properties into mosquito populations, which can lead to dramatic decreases in the incidence of the disease. In light of these promising results, there is a crucial need to better understand the factors affecting the success of such strategies, in particular the choice of *Wolbachia* strain for field releases and the potential for evolutionary changes. Here, we characterized two variants of a *Wolbachia* strain used for dengue control that differ at the genomic level and in their ability to replicate within the mosquito. We also found no evidence for the evolution of the symbiont within the 2 years following its deployment in Malaysia. Our results have implications for current and future *Wolbachia*-based health interventions.

## INTRODUCTION

Aedes aegypti mosquitoes are the primary vectors of dengue, a neglected viral disease ranked by WHO among the top 10 global health threats, with 50 to 100 million clinically apparent cases and half a million hospitalizations for severe disease every year (1). Current control methods based on insecticide fogging for mosquito suppression have failed to halt the continued expansion in range and incidence of dengue, and rising levels of insecticide resistance mean that there is a pressing need for innovative approaches. *Wolbachia* species are maternally inherited symbiotic bacteria found in many insect species, but not naturally in *Ae. aegypti* (2); however, following lab transfer into this species some *Wolbachia* strains can efficiently block dengue transmission (3–6) by causing perturbations in various cellular pathways, including lipid transport (7).

*Wolbachia* strains *w*Mel from Drosophila melanogaster and *w*AlbB from Aedes albopictus have both been shown to spread to and remain at a high frequency in *Ae. aegypti* populations following releases of *Ae. aegypti* at a comparatively modest scale and duration without needing continuous reintroduction (8–11). These strains have a self-spreading capability using a form of reproductive manipulation known as cytoplasmic incompatibility (CI), whereby the progeny of *Wolbachia*-carrying males and *Wolbachia*-free females die, while the reverse cross is fertile, giving an advantage to *Wolbachia*-carrying females. Both strains have been shown to efficiently reduce dengue transmission, providing a safe, sustainable, cost-effective, and eco-friendly biocontrol tool that holds great promise for reducing the global burden of dengue (8, 11–14).

Since releases of *Ae. aegypti* carrying *w*AlbB (6) were carried out in Malaysia at sites around Kuala Lumpur that were previously hot spots for dengue transmission, dengue has been substantially decreased (8). When larvae develop under high-temperature regimens with diurnal peaks around 37°C, *w*AlbB is more stable than *w*Mel, maintaining a higher density, high maternal transmission, and efficient dengue transmission blocking (6, 15–18). The fitness cost of *w*AlbB in *Ae. aegypti* is higher than that of *w*Mel in lab assays, mainly due to slightly reduced adult longevity (6) and reduced fertility and fecundity of adult females produced from quiescent eggs (19). *Wolbachia* fitness costs negatively affect population dynamics, raising the threshold frequency that must be exceeded for CI-mediated spread to occur (20–22) and for *Wolbachia* to remain at stable high frequency after introduction, as occurred with *w*AlbB at a number of sites in Malaysia (8). Several independent transinfections of *w*AlbB from *Ae. albopictus* have been generated in *Ae. aegypti* through microinjection (6, 23, 24), and two of these have been released in natural populations (8, 25). While the transinfections originate from different geographic locations, it is unclear if there are genetic or phenotypic differences between them.

The effectiveness of *Wolbachia* interventions against dengue could be compromised in the longer term by evolutionary changes in the *Wolbachia* or mosquito genome (26). Virus transmission blocking could be reduced over time if mosquito-*Wolbachia* coevolution results in lower *Wolbachia* density overall or more restricted tissue distribution to the ovaries and testes. The *w*AlbB-associated reduced hatch rate of stored *Ae. aegypti* eggs could also be ameliorated by natural selection (27); if this selection acts specifically at the egg stage and does not impact the dengue transmission-blocking phenotype, it would be advantageous overall for implementation of the strategy. No obvious phenotypic changes have been observed in *w*AlbB to date in field populations of *Ae. aegypti* (18), but longer-term monitoring is required.

The primary aim of this study was to sequence the genome of the *w*AlbB strain released in Malaysia. This is useful for several reasons: to be able to ascertain whether this *w*AlbB has any unique genomic features relative to a previously published *w*AlbB genome, to be able to track genomic evolution that may occur in the field that could potentially compromise the effectiveness of the dengue intervention, and to allow for the creation of molecular assays for distinguishing this variant from the naturally occurring *w*AlbB present in *Ae. albopictus*, which will be useful in *Wolbachia* frequency monitoring, since both mosquito species are present at the intervention sites. Other aims were to compare the impacts of different *w*AlbB infections on *Wolbachia* density, egg quiescence, and responses to heat.

## RESULTS

### Comparative genomics of *w*AlbB genomes.

**FIG 1:**
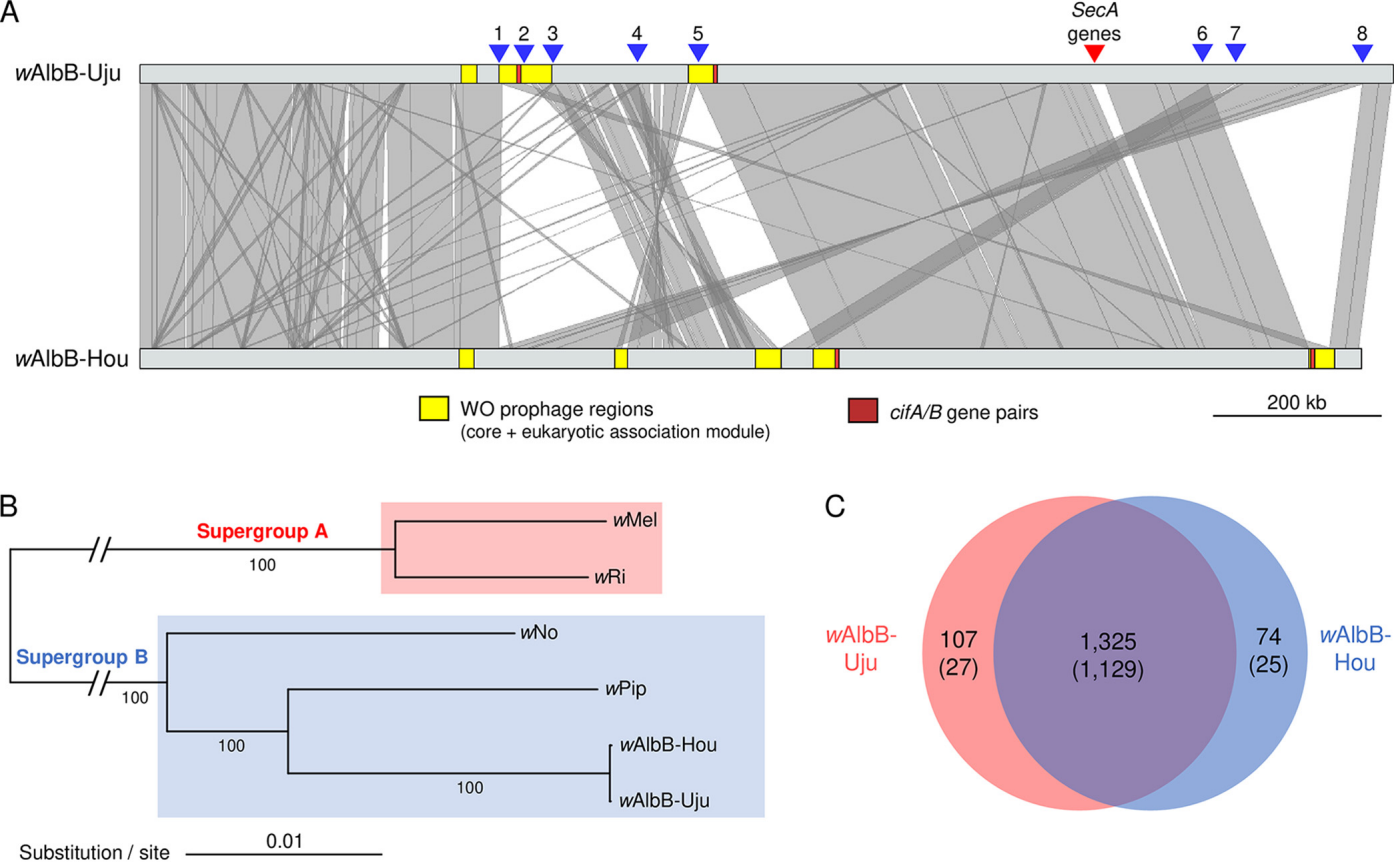
Comparative analysis and phylogeny of *w*AlbB genomes. (A) Genome-wide synteny. Gray areas between genomes indicate similarities based on a megablastn comparison. BLAST hits and contigs of <3,000 bp were excluded from the figure, and genomes were rotated to start at the *DnaA* gene to improve visualization. Triangles indicate major chromosomal breakpoints (blue) and two syntenic putatively horizontally transferred *SecA* genes. (B) Maximum likelihood phylogeny using a concatenated alignment of 614 orthologous genes. Node labels are bootstrap supports calculated from 1,000 replications. (C) Venn diagram showing numbers of orthologues shared between *w*AlbB genomes. (Numbers in parentheses exclude transposable elements.)

We generated a new circular genome assembly from an Indonesian *w*AlbB that was previously isolated from the *Ae. albopictus* line UJU (origin, Sulawesi Island) and subsequently transferred into *Ae. aegypti* in 2015 using embryo microinjection (6). We compared this new assembly to the publicly available *w*AlbB reference circular genome (Fig. 1A), which is derived from an *Ae. albopictus* mosquito line caught in Houston, TX, USA, in 1986 and has subsequently been maintained in the *Ae. albopictus* Aa23 cell line (28, 29). The Indonesian *w*AlbB genome is 1.52 Mb in size which is slightly longer than the reference *w*AlbB genome (1.48 Mb); however, the two variants show 99.96% sequence identity across their genomes, and the same numbers of single-copy conserved orthologues were found in both (Table 1). In light of the genomic differences described below, we will refer to *w*AlbB-Hou and *w*AlbB-Uju to designate the reference variant from the Texas variant and Indonesian variant, respectively.

**TABLE 1:** Genome features of sequenced wAlbB genomes

Parameter	Genome feature for:	
	*w*AlbB-Uju	*w*AlbB-Hou
Genbank accession no.	CP102671	CP031221.1
Geographical origin	Indonesia (Sulawesi)	USA (TX)
Assembly size (bp)	1,523,308	1,484,007
GC content (%)	34.4	34.4
No. of:		
Genes	1,476	1,434
CDS	1,435	1,393
rRNAs	3	3
tRNAs	34	34
BUSCO analysis, no.^*a*^		
Complete	328	328
Fragment	9	9
Missing	95	95

a The BUSCO analysis was run against the alphaproteobacteria_odb10 database.

**FIG 2:**
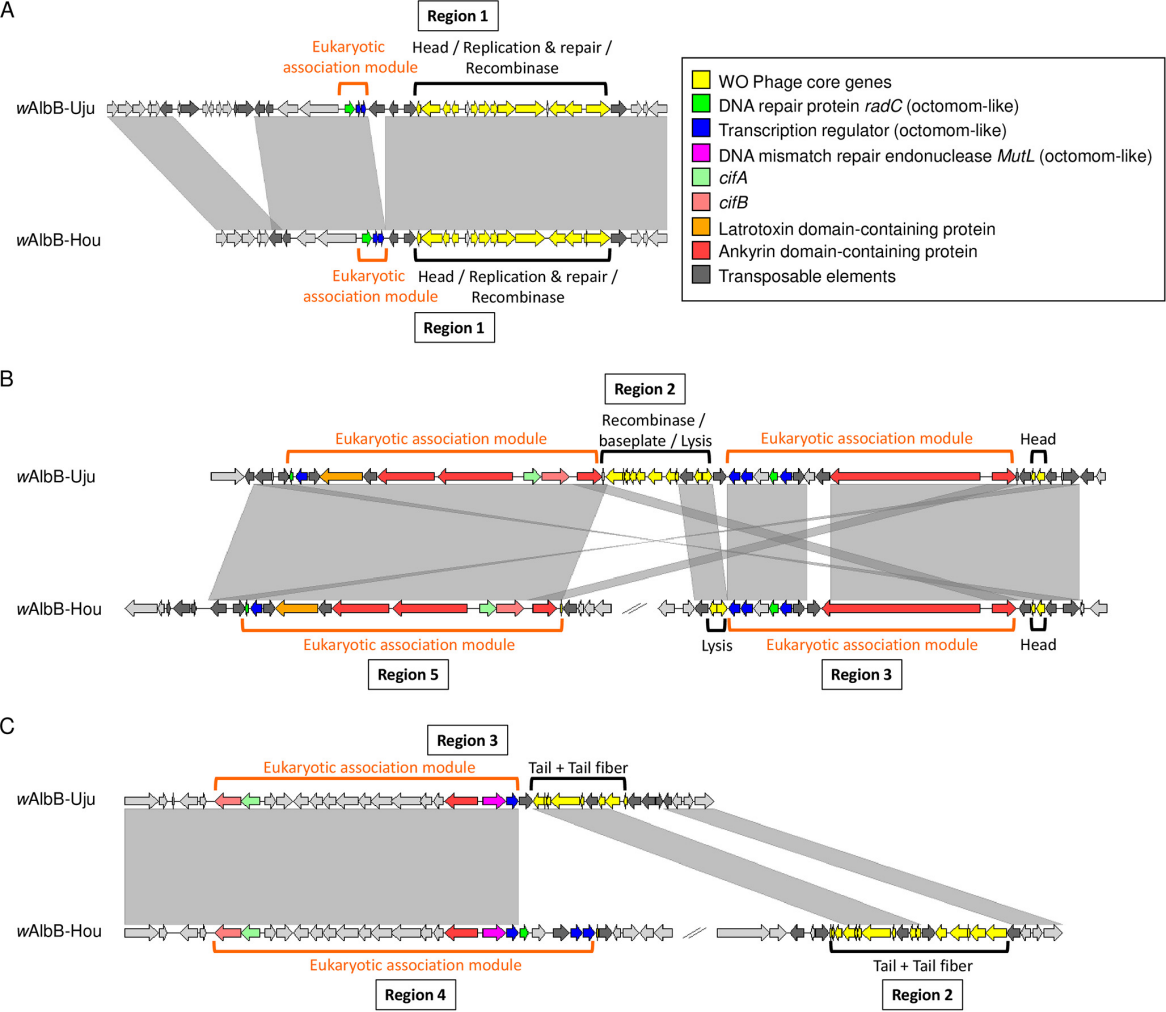
Synteny of WO prophage regions between *w*AlbB-Uju and *w*AlbB-Hou genomes. Panels depict the comparisons between the three prophage regions of *w*AlbB-Uju and those of *w*AlbB-Hou as illustrated in Fig. 1A. Gray areas indicate similarities based on megablastn comparisons. BLAST hits of <2,000 bp were excluded from the figure to improve visualization.

The *w*AlbB-Uju variant clusters with the reference *w*AlbB-Hou genome into a monophyletic clade within *Wolbachia* supergroup B (Fig. 1B). However, despite the strong phylogenetic relatedness, *w*AlbB-Uju displays striking differences in genome synteny, with eight major chromosomal breakpoints between the two *w*AlbB variants (Fig. 1A), all supported by long-read sequencing data (see Fig. S1A to G in the supplemental material). Repeat elements (transposases, reverse transcriptases, and related pseudogenes) were always located on the breakpoint site or right next to it, suggesting that repeat regions have played a major role in genome rearrangement among *w*AlbB variants (Fig. S1). There are three incomplete WO prophage regions in the *w*AlbB-Uju genome, indicating ancient WO phage infections. The structural and nonstructural modules (head, tail, baseplate, replication, and eukaryotic modules) are split between the different regions, suggesting no active phage replication (Fig. 2). Moreover, essential genes of the phage head, tail, and baseplate are missing, indicating that the production of phage particles is impaired (Table 2). Core phage genes are present in single copies, except for the recombinase and phospholipase D, which are both present in two copies with high sequence divergence, suggesting that the *w*AlbB genome may have been colonized in the past by more than one WO phage (Table 2). The *w*AlbB-Hou genome also carries prophage regions with sequence similarities to those of *w*AlbB-Uju, but these have rearranged into five different regions (Fig. 1A). Two pairs of the cytoplasmic incompatibility-inducing genes *cifA* and *cifB* are located within *w*AlbB-Uju’s prophage regions and are identical to those of *w*AlbB-Hou. The two gene pairs are related to type III *cif* homologues for one pair and type IV homologues for the other pair as defined in previous studies (30, 31).

**TABLE 2:** Presence/absence of WO phage core genes in prophage regions

Module	Gene product annotation (locus tag in *w*AlbB-Hou)	Presence in^*a*^:	
		*w*AlbB-Uju	*w*AlbB-Hou
Head	Ankyrin repeat protein (DEJ70_04885)	+	+
	Terminase large subunit (DEJ70_04880)	+	+
	Putative terminase small subunit (DEJ70_04890)	+	+
	gpW	−	−
	Portal	−	−
	Minor capsid C	−	−
	Head decoration protein D	−	−
	Major capsid E (DEJ70_06645)	+	+

Connector/baseplate	gpFII (DEJ70_06650)	+	+
	Minor tail protein Z	−	−
	Collar	−	−
	gpV	−	−
	PAAR	−	−
	gpW	+	−
	gpJ	+	−
	Gpl	+	−

Tail	Tail sheath (DEJ70_05870–DEJ70_05880)	+	+
	Tail tube (DEJ70_05865)	+	+
	gpG/GT (DEJ70_05855/unannotated downstream ORF)	+	+
	Tape measure (DEJ70_05850)	+	+
	gpU (DEJ70_05845)	+	+
	gpX (DEJ70_05840)	+	+
	Late control D (DEJ70_05835)	+	+

Tail fiber	Baseplate wedge 3 tail fiber network	−	−
	Receptor-binding protein (DEJ70_05895–DEJ70_05900)	−	+
	Tail fiber assembly chaperone (DEJ70_05890)	−	+
	Receptor-binding protein or tail fiber (DEJ70_05885)	+	+
	Tail fiber assembly chaperone	−	−

Recombinase	Recombinase (DEJ70_04945)	++	+

Replication and repair	Holliday junction resolvase (DEJ70_04895)	+	+
	AAA family ATPase (DEJ70_04910)	+	+
	AAA family ATPase (DEJ70_04920)	+	+
	RNA_polymerase_sigma_factor (DEJ70_04905)	+	+
	DNA primase (DEJ70_04925)	+	+

Putative lysis	Patatin-like phospholipase (DEJ70_06735)	+	+
	Phospholipase D (DEJ70_06740, DEJ70_06035)	++*	++*

a A gene is either present (+) or absent (−). Differences between variants are highlighted in gray. An asterisk indicates that one copy of the phospholipase D is located outside prophage regions.

In addition to chromosomal rearrangements, we found noticeable differences in gene content between the *w*AlbB-Hou and *w*AlbB-Uju genomes (Fig. 1C and Table S1), with ~70% of the differences involving repeat elements (transposases, reverse transcriptases, and related pseudogenes). Excluding repeat elements, *w*AlbB-Uju harbors 27 genes that are either absent from or pseudogenized in *w*AlbB-Hou, while on the other hand, *w*AlbB-Hou carries 25 genes not found or pseudogenized in *w*AlbB-Uju. Some of this variation is located within and around prophage regions, where *w*AlbB-Uju and *w*AlbB-Hou genomes have lost different core and accessory phage genes (Fig. 2, Table 2, and Table S1). For instance, one of the *w*AlbB-Uju phage eukaryotic modules lacks two copies of a putative transcriptional regulator and one copy of a DNA repair protein which are homologues of genes in an eight-gene locus known as Octomom thought to influence *Wolbachia* proliferation in *w*Mel-like strains (32–34) (Fig. 2C). Interestingly, *w*AlbB-Uju also carries two syntenic proteins (L3551_05645 and L3551_05650) with homologies to arthropod protein translocase subunit *SecA* genes that are absent in *w*AlbB-Hou (Fig. 1A, Fig. S1H, and Table S1) but present in other *Wolbachia* strains (35). The two genes are of unusual length for *Wolbachia* genes (4,491 and 11,664 bp, respectively, versus 900 bp on average for other *Wolbachia* genes). They branch with a few other *Wolbachia* homologues within arthropod lineages with no closely related bacterial homologues, indicating two independent horizontal transfers from arthropods to *Wolbachia* in the case of L3551_05645 and at least one such event for L3551_05650 (Fig. S2). Finally, several genes differed between *w*AlbB variants due to pseudogenization by the insertion of a transposase. For example, a homologue of the *Wolbachia* surface protein gene *wspB* is truncated in *w*AlbB-Hou, while a full-length version of the gene is present in *w*AlbB-Uju (Table S1). Except for transposable elements, the absence of genes in one variant was also confirmed by the absence of Illumina reads mapping to the genome of the other variant (Fig. S3).

Numerous single nucleotide polymorphisms (SNPs) and small indels between the two *w*AlbB genomes were also identified (Table 3 and Table S2). Around half of the SNPs between *w*AlbB-Uju and *w*AlbB-Hou are nonsynonymous, of which some are located within ankyrin repeat domain-containing genes as well as genes potentially involved in transcription and RNA processing (e.g., genes encoding sigma factor RpoD [36], transcription elongation factor NusA [37], RNA polymerase subunits alpha and beta, and ribonucleases E and D [38]), protein synthesis (e.g., genes encoding ribosomal proteins and translational GTPase TypA [39]), cell wall synthesis and remodeling (e.g., genes encoding *N*-acetylmuramoyl-l-alanine amidase, d-alanyl-d-alanine carboxypeptidase, M23 family peptidase, UDP-*N*-acetylmuramate dehydrogenase) (40, 41), and stress response (e.g., genes encoding the heat shock proteins ATP-dependent Clp endopeptidase [42] and DegQ endoprotease [43]).

**TABLE 3:** Summary of the SNP analysis

Genome by Illumina reads	No. of SNPs in genome used as reference for SNP calling	
	*w*AlbB-Uju	*w*AlbB-Hou
*w*AlbB-Uju	0	130
*w*AlbB-Uju-MC	0	134
*w*AlbB-Hou	141	0

### *w*AlbB variants display background-dependent differences in density.

**FIG 3:**
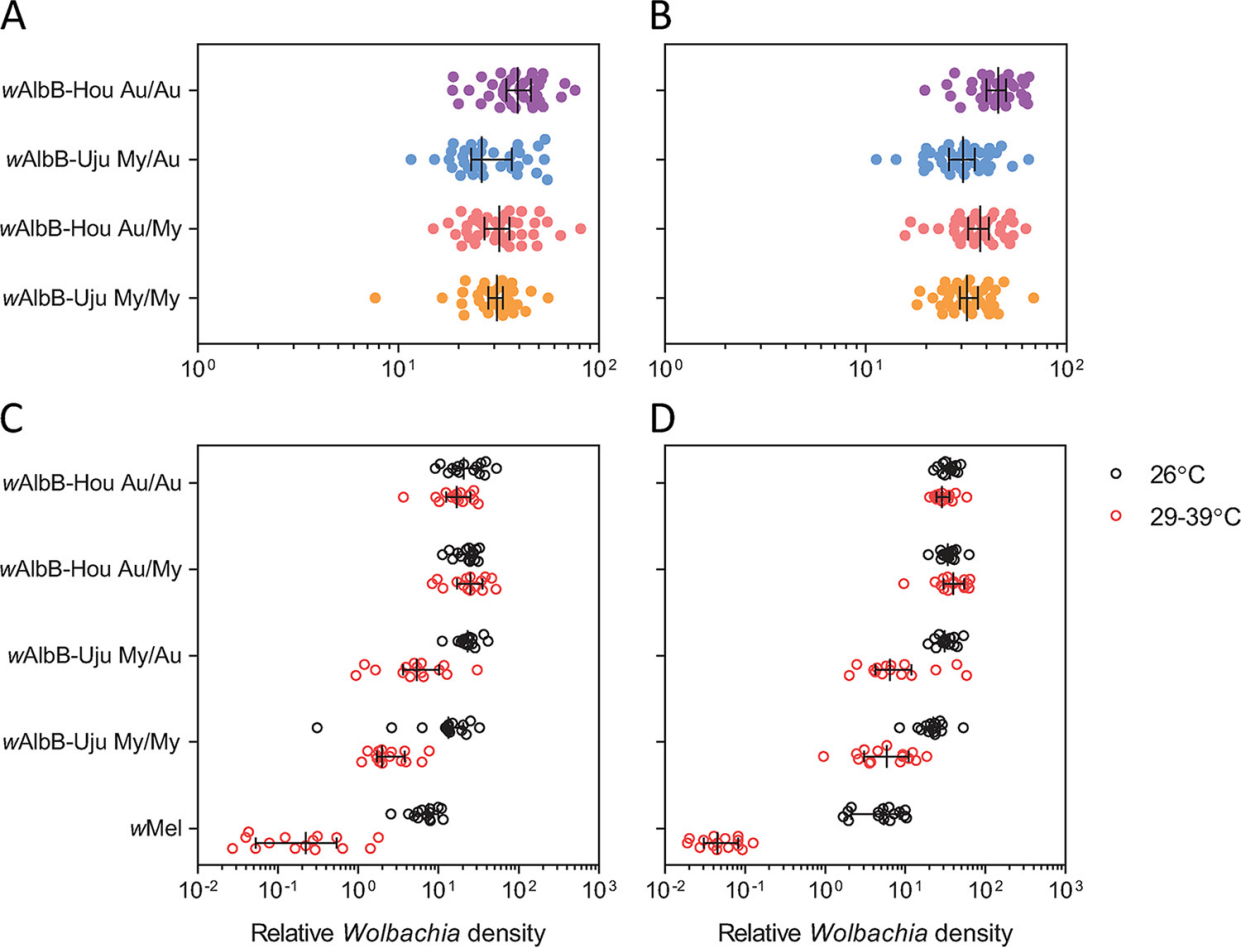
Differences in density between *w*AlbB variants. (A and B) Female (A) and male (B) *Wolbachia* density in reciprocally backcrossed Aedes aegypti populations. Populations have different combinations of *Wolbachia* infection type/mitochondrial haplotype (*w*AlbB-Hou and Au or *w*AlbB-Uju and My) and nuclear background (Au or My). Data from two replicate populations were pooled for visualization. (C and D) *Wolbachia* density in (C) females and (D) males following exposure to cyclical heat stress during the egg stage. Eggs were exposed to cyclical temperatures of 29 to 39°C for 7 days (red circles) or held at 26°C (black circles). Each point represents the relative density for an individual averaged across 2 to 3 technical replicates. Medians and 95% confidence intervals are shown in black lines. Data for *w*AlbB-I and *w*Mel have also been included from reference 44.

To determine potential effects of *w*AlbB variation on phenotype, we generated *Ae. aegypti* populations with different *Wolbachia* infection types (*w*AlbB-Hou, *w*AlbB-Uju, or uninfected) and mosquito backgrounds (Australian [Au] or Malaysian [My]) through reciprocal backcrossing (44). *Wolbachia* density was influenced by *w*AlbB variant in both sexes (Table S3), with *w*AlbB-Hou individuals having higher *Wolbachia* densities than *w*AlbB-Uju individuals (Fig. 3A and B). We found no clear effect of nuclear background for either sex, but there was a significant interaction between nuclear background and *w*AlbB variant in males (Table S3). In the Australian background, *w*AlbB-Hou had a higher density than *w*AlbB-Uju (general linear model [GLM]; females, *F*_1, 76_ = 13.792, *P* < 0.001; males, *F*_1, 73_ = 27.401, *P* < 0.001), but there were no significant differences between variants in the Malaysian background (females, *F*_1, 76_ = 0.982, *P* = 0.325; males, *F*_1, 76_ = 2.225, *P* = 0.140).

To test the stability of *w*AlbB variants at high temperatures, we measured *Wolbachia* densities in adults after eggs were exposed to cyclical heat stress (29 to 39°C) or held at 26°C for 1 week. *Wolbachia* density was influenced by *w*AlbB variant and temperature treatment, with significant interactions between *w*AlbB variant and nuclear background as well as *Wolbachia* variant and temperature (Table S4). When the *w*AlbB-Hou and *w*AlbB-Uju variants were tested separately, we found no effect of temperature or nuclear background in either sex (all at *P* > 0.141) for *w*AlbB-Hou, indicating that this infection is stable under heat stress (Fig. 3C and D). In contrast, *w*AlbB-Uju density was lower in the heat stress treatment (females, *F*_1, 56_ = 51.940, *P* < 0.001; males, *F*_1, 56_ = 61.814, *P* < 0.001) and in the Malaysian background (females, *F*_1, 56_ = 12.831, *P* = 0.001; males, *F*_1, 56_ = 4.549, *P* = 0.037). Across both sexes and backgrounds, median *w*AlbB-Uju density under cyclical heat stress decreased by 80.4%, compared to 8.1% for *w*AlbB-Hou and 98.7% for *w*Mel.

### Quiescent egg viability depends on mosquito nuclear background and *w*AlbB infection.

**FIG 4:**
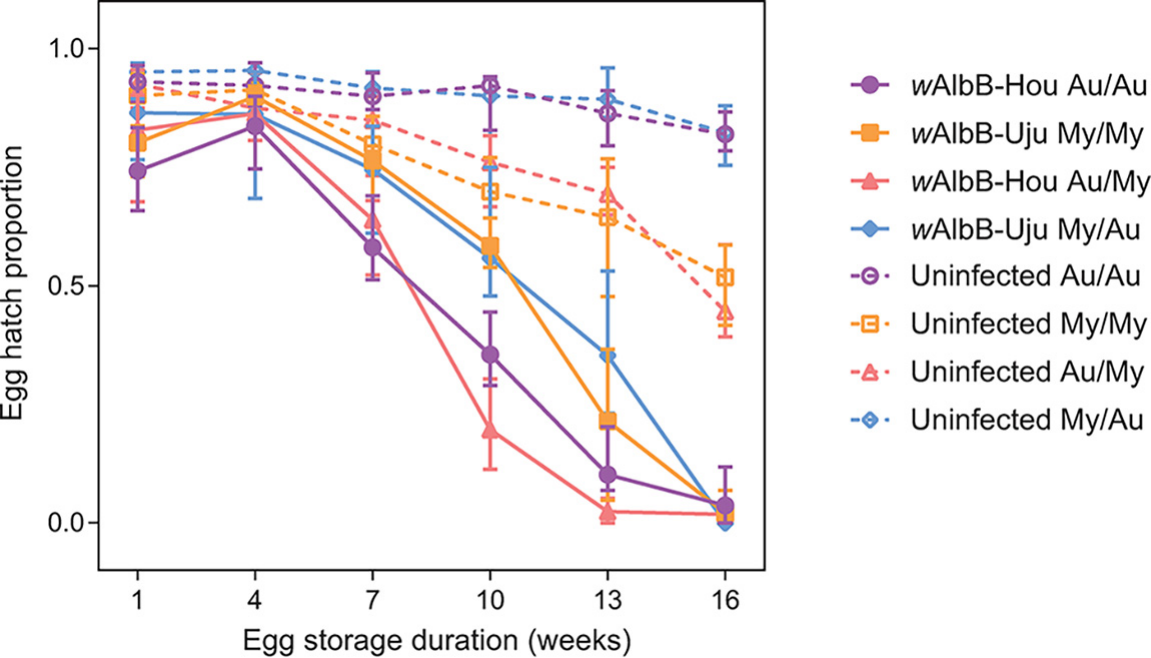
Quiescent egg viability of reciprocally backcrossed Aedes aegypti populations. Populations have different combinations of *Wolbachia* infection type (*w*AlbB-Hou, *w*AlbB-Uju, or uninfected), mitochondrial haplotype (Au or My), and nuclear background (Au or My). Data from two replicate populations were pooled for visualization. Symbols show median egg hatch proportions, while error bars show 95% confidence intervals. Data for the Au/Au and Au/My populations have also been included from reference 44.

Stored eggs from populations with different combinations of *w*AlbB infection type (*w*AlbB-Hou, *w*AlbB-Uju, or uninfected) and mitochondrial haplotype (Au or My), and nuclear background (Au or My) were hatched every 3 weeks to determine quiescent egg viability. *w*AlbB infection greatly reduced quiescent egg viability in all four combinations of background and mitochondrial haplotype (Fig. 4). By week 16, hatch proportions for *w*AlbB-infected populations approached zero while hatch proportions for uninfected populations exceeded 40%. In uninfected populations, we found significant effects of egg storage duration and nuclear background on egg hatch proportions (Table S5). Eggs with an Australian background had higher hatch proportions (median, 0.823) than eggs with a Malaysian background (median, 0.503) by the end of the experiment. In *w*AlbB-infected populations, we found significant effects of egg storage duration, squared egg storage duration, nuclear background, and replicate population (Table S5). Although populations carrying *w*AlbB-Uju had higher overall hatch proportions than *w*AlbB-Hou populations in both backgrounds (Fig. 4), effects of *w*AlbB variant were not significant (Table S5). We had low power to detect *w*AlbB variant effects in this analysis due to nesting of the replicate population within *w*AlbB variant.

### A multiplex PCR for diagnostics of *w*AlbB variants.

**FIG 5:**
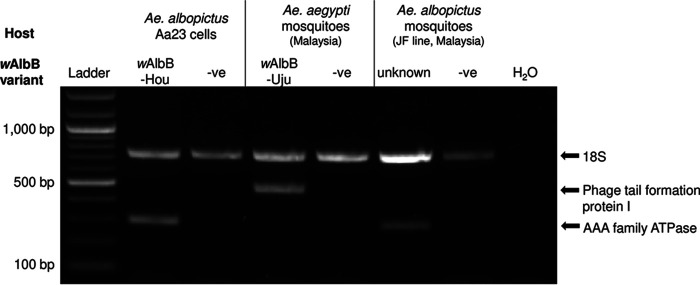
*w*AlbB variant-specific multiplex PCR. DNA was extracted from approximately 1 × 10^6^ cells and five mosquito females for the Aa23 and mosquito samples, respectively. -ve, *Wolbachia*-negative controls.

Given the genomic differences observed between the *w*AlbB genomes, we developed a PCR allowing the distinction between the *w*AlbB-Hou and -Uju variants in *Ae. aegypti* and *Ae. albopictus*. From our gene content analysis, we selected an AAA family ATPase protein and the phage tail formation protein I as markers to distinguish the two *w*AlbB variants. Primers for these markers were designed and pooled with primers amplifying the 18S mosquito control gene into a multiplex PCR. *w*AlbB-Hou in the Aa23 *albopictus* cell line and *w*AlbB-Uju in our *Ae. aegypti* mosquito lab line displayed the expected band profiles and were clearly distinguishable on the agarose gel (Fig. 5). Moreover, the *w*AlbB variant infecting a Malaysian line of *Ae. albopictus* mosquitoes (JF) displayed identical bands to *w*AlbB-Hou variant.

### No evidence for evolution of the *w*AlbB-Uju genome following field release.

Using the *w*AlbB-Uju assembly as a reference, we mapped the sequencing data generated from a *w*AlbB-infected *Ae. aegypti* colony, *w*AlbB-Uju-MC (Mentari Court site), which was isolated from the field 2 years following field releases of wAlbB-Uju in Malaysia (18). All genome positions of the *w*AlbB-Uju assembly were covered by read data from *w*AlbB-Uju-MC, with no drastic drop in sequencing depth, suggesting that no gene was lost since field releases (Fig. S3A). Additionally, no SNPs were detected; therefore, we conclude that there is little evidence for genomic changes that may have occurred following the introduction of *w*AlbB-Uju in the field.

## DISCUSSION

Here, we uncovered major genomic differences between closely related variants of the *w*AlbB *Wolbachia* strain and showed these differences correlate with variation in symbiont density. *w*AlbB diversity has commonly been investigated using a limited number of markers, such as the *wsp* gene and multilocus sequence typing (MLST) genes, with little to no variation observed between isolates from different locations (45–47). Whole-genome sequencing provides a higher resolution and has revealed significant genomic differences between closely related *Wolbachia* strains (48–50). Using this method, we demonstrated the existence of at least two types of *w*AlbB variants that differ in genome synteny and gene content. Further sampling of *w*AlbB genomic diversity will provide new insights into the evolution of this symbiont lineage and may help unravel the colonization history of its native host, *Ae. albopictus*, across continents (51).

Importantly, there are differences in symbiont density between the *w*AlbB-Hou and *w*AlbB-Uju variants. Although only one isolate representative of each variant was characterized here, these results suggest that variant-specific genetic determinants may be driving some of the differences in density. It is also possible that differences in mitochondrial haplotypes contributed to some of the variation in symbiont density since mitotypes were not cross-factored with the *w*AlbB variants. The genomes of *w*AlbB-Hou and *w*AlbB-Uju variants differed in the presence/absence of 52 genes (excluding transposable elements) and on ~130 SNPs, 50% of which were nonsynonymous. How much of this variation contributes to differences in symbiont density remains to be investigated, and further phenotypic characterization of new variants will help shorten the list of candidate genes since there is currently no transformation system in *Wolbachia* for functional validation. Interestingly, some of the genomic differences are located within prophage regions. It has been hypothesized that WO phage replication lowers *Wolbachia* density (52); however, we only found incomplete prophage regions, suggesting that active phage mobilization is unlikely to occur in *w*AlbB. Alternatively, variation in the expression of phage accessory genes could be responsible for differences in density. Indeed, *w*AlbB-Hou carries additional copies of a DNA repair protein gene, *radC*, and a gene coding for a putative transcriptional regulator in its phage eukaryotic module. Homologues of these genes in *w*Mel-like strains, WD0507 and WD0508, are part of a 20-kb region called Octomom. Octomom is to date the only genetic determinant shown to influence *Wolbachia* proliferation in a copy-number-dependent manner (32). Both complete loss of the region and amplification have been associated with symbiont overproliferation, which in turn has negative effects on host life span (33, 34).

Density variation could also stem from the way *w*AlbB variants interact with the host. *Wolbachia* genomes commonly harbor an array of ankyrin domain-containing genes, which are predicted to be involved in protein-protein interactions as well as secretion systems that may allow the export of bacterial effectors into the host cell cytoplasm (53, 54). Several ankyrin domain-containing genes are among the candidate genes that differ between *w*AlbB variants. Additionally, *w*AlbB-Uju harbors two syntenic proteins showing homologies to arthropod protein translocase subunit *SecA* genes. Homologues were previously found in other *Wolbachia* strains (35), and their phylogenetic distribution points toward an acquisition through horizontal transfer from a eukaryotic host. *SecA* proteins are involved in the transport of bacterial and ER-exported proteins (55), suggesting that the *w*AlbB variants may differ in the way they interact with the host cell. Interestingly, *w*AlbB variants also differed in a homologue of the *Wolbachia* surface protein gene *wspB*. *wspB* is pseudogenized in *w*AlbB-Hou, and this variant maintains higher densities under a high-temperature cycle than *w*AlbB-Uju, which carries a full-length version of the gene. This is consistent with two recent studies showing that, among variants of the *Wolbachia* strain *w*Mel, pseudogenization of the *wspB* gene is associated with variation in symbiont density and maternal transmission and that the magnitude of this effect can vary with both temperature and host background (56, 57). Finally, *w*AlbB variants differed in a number of housekeeping genes involved in essential functions, such as DNA replication, RNA processing, translation, or cell wall biogenesis that may contribute to the variation in symbiont density, as well as in heat shock response genes that could potentially control their different degrees of tolerance of heat stress.

The *w*AlbB-Hou variant used in our phenotypic assays originates from the same *Ae. albopictus* line as the *w*AlbB-Hou reference genome; however, it was transferred to and maintained in *Ae. aegypti* for 15 years before being sequenced (see Materials and Methods) and only differs from the reference genome by four SNPs located in a putative ferric transporter (DEJ70_01985), an ankyrin repeat domain-containing protein (DEJ70_06660), a hypothetical protein (DEJ70_02515), and an intergenic region (44). While it was initially hypothesized that these changes occurred following the transfer to *Ae. aegypti* (44), we detected the same four SNPs when comparing *w*AlbB-Uju and *w*AlbB-Uju-MC to the reference *w*AlbB-Hou genome. Therefore, it is more likely that the changes occurred in *w*AlbB-Hou in the Aa23 cell line rather than in the *Ae. aegypti* mosquito line. All else being equal, this means that the *w*AlbB variants used in the present genome comparisons and phenotypic assays are directly comparable, except for the fact that *w*AlbB-Hou and *w*AlbB-Uju are identical at these four nucleotides in the phenotypic characterization.

The phenotypic differences detected impact on the relative ability of these variants to spread through *Ae. aegypti* populations, and their efficacy for dengue control programs. The decline in hatch rates over time of dried quiescent eggs is an important component of the fitness costs of *w*AlbB in this host (19). In areas where a high proportion of larval sites are temporary and experience intermittent inundation, dry eggs are often in quiescence for extended periods, and thus the fitness cost of *w*AlbB will be higher relative to *Wolbachia*-free wild-type counterparts than when larvae develop in permanent breeding sites such as water storage tanks. This factor will increase the threshold population frequency that must be exceeded for *Wolbachia* to spread/remain stable in the population. The higher, background-dependent negative impact of the *w*AlbB-Hou on quiescent egg hatch rates means that this variant will be predicted under some backgrounds/ecological conditions to spread less efficiently than *w*AlbB-Uju. Conversely the apparent slightly higher tolerance of *w*AlbB-Hou for very high temperatures during egg storage may also impact relative spread dynamics since maintenance of higher density of *w*AlbB-Hou may ultimately impact maternal transmission rates and virus inhibition. In the hottest climates in which *Ae. aegypti* occurs, *w*AlbB-Hou may prove to be a better option for dengue control than *w*AlbB-Uju. However, more data are needed under a variety of conditions and over multiple generations and life stages to test the relative impacts of temperature in more detail.

Previously, we found negligible changes in the *w*AlbB-Hou genome following transfer from its native host, *Ae. albopictus*, to *Ae. aegypti* (44), suggesting that there is little selective pressure to adapt to this new host, at least under laboratory conditions. This is in line with little genomic changes observed following artificial transfers of multiple *Wolbachia* strains between *Drosophila* species (58). Here, we found no evidence of wAlbB-Uju genome evolution 2 years after its introduction in a field population of *Ae. aegypti*, which supports our earlier results showing stable density and antiviral effects using the same field-caught mosquito colony (18). While we found striking differences between closely related *w*AlbB genomes, this suggests that such genomic changes may only occur over long periods of time, thus maintaining the efficacy of *Wolbachia*-based disease control over many years. However, further monitoring of *Wolbachia* genomes at release sites is still required to confirm if this holds true over the longer term. Nevertheless, similar results were observed for the *w*Mel genome, which remained relatively stable almost a decade following the introduction of *Wolbachia*-infected *Ae. aegypti* mosquitoes in Australia (59–61). *w*Mel and *w*MelPop-CLA infections in *Ae. aegypti* also show few long-term phenotypic changes following transinfection (10, 62).

## MATERIALS AND METHODS

### *Wolbachia* purification.

In order to generate Illumina sequencing data for both the reference *w*AlbB-Uju genome and its field-caught counterpart, *w*AlbB-Uju-MC, *Wolbachia* was purified from whole mosquitoes. *w*AlbB-Uju-MC-infected mosquitoes were used after three generations spent in the lab since field collection in Mentari Court. For each genome, around 400 mosquitoes were collected into a 50-mL Falcon tube and snap-frozen at −20°C for 10 min. Mosquitoes were then surface sterilized for 3 min in 50% bleach, followed by 3 min in 70% ethanol, and rinsed 3 times with sterile water. Mosquitoes were then manually homogenized with 3-mm glass beads in 40 mL of Schneider’s medium by shaking and further homogenized with a generic tissue lyzer after transferring the homogenate into 2-mL tubes with 1-mm beads. Homogenates were centrifuged at 2,000 × *g* for 2 min to remove tissue debris, and the supernatant was sequentially filtered through 5-, 2.7-, and 1.5-μm-pore sterile filters. The filtrate was aliquoted in Eppendorf tubes and centrifuged at 18,500 × *g* for 15 min to pellet bacteria. The supernatant was discarded, Schneider’s medium added, and the previous centrifugation step repeated once. The bacterial pellet was resuspended in Schneider’s medium and treated with DNase I at 37°C for 30 min to remove host DNA. Following digestion, samples were centrifuged at 18,500 × *g*, the supernatant discarded, and the DNase inactivated at 75°C for 10 min. Finally, bacterial pellets were pooled into one tube and DNA extracted with the Gentra Puregene tissue kit (Qiagen) with resuspension of the DNA pellet in 100 μL of nuclease-free water.

For long-read sequencing of the *w*AlbB-Uju genome, DNA was extracted from dissected ovaries pooled from 43 female mosquitoes, and no *Wolbachia* purification was performed.

### Whole-genome sequencing and genome assembly.

For Illumina sequencing, DNA libraries were prepared using the Kapa LTP library preparation kit (Kapa Biosystems; Roche7961880001) and sequenced on the Illumina MiSeq platform with the MiSeq reagent kit v.3 to generate 2 × 150-bp reads. Raw reads were demultiplexed using bcl2fastq, and adapters were trimmed with Trimmomatic v.0.38.0 (63). To generate long reads for *w*AlbB-Uju, the library was prepared with a rapid sequencing kit (SQK-RAD0004) from Oxford Nanopore Technologies (ONT) using the ultralong-read protocol with some modifications (64). In short, the library was made from 4 μg genomic DNA (gDNA) conditioned with 0.02% Triton X-100. DNA was tagmented with 1.5 μL transposome mix, and 1 μL of rapid sequencing adaptor was then attached to DNA ends. The library was loaded on a MinION flow cell (R9.4.1, FLO-MIN106) and sequenced for 48 h on a GridION instrument controlled by the MinKNOW software v.22.03.4 (ONT). Base-calling and demultiplexing were performed within MinKNOW using Guppy v.4.0.11. ONT adapters were removed with Porechop v.0.2.4 (65). Host reads were filtered out by mapping the Illumina and Nanopore reads against the *Ae. aegypti* reference assembly (GenBank accession no. GCF_002204515.2) using Bowtie2 v.2.4.2 (66) and Minimap2 v.2.23 (67), respectively. Unmapped Nanopore reads were first assembled using Canu v.2.2 (68), which produced a noncircular 1,451,763-bp contig corresponding to the *Wolbachia* genome. In order to complete the assembly, both decontaminated Illumina and Nanopore reads were then assembled using the Unicycler hybrid assembly pipeline with the draft Canu assembly as a guide (“–existing_long_read_assembly” option) (69). Contigs were visualized and BLAST searched against several *Wolbachia* genomes in Bandage (70), and non-*Wolbachia* sequences were discarded from the assembly. The final assembly showed mean sequencing depths of 100× and 17× for Illumina and Nanopore reads, respectively.

### Comparative genomics.

Long reads from *w*AlbB-Uju (SRA accession no. SRR21023725) and *w*AlbB-Hou (SRA accession no. SRR7784287) were used to analyze major chromosomal breakpoints between the two variants by mapping the reads onto the *w*AlbB-Uju genome with Minimap2 v.2.23 and visualizing the alignments in IGV v.2.12.3 (71). The *w*AlbB-Uju and *w*AlbB-Hou genomes were all reannotated using Prokka v.1.14.6 (72) prior to the gene content analysis. Roary v.3.13.0 (73) was then used to determine the core and accessory genomes with a 95% identity threshold. The Roary output was manually curated to fix issues with pseudogenes in the accessory genomes by visualizing the genome annotations in the Artemis genome browser v.16.0.0 (74). Indeed, putative pseudogenes were often split into two or more open reading frames due to internal stop codons, frameshifts, or the insertion of transposases within the gene. This caused Roary to wrongly categorize different parts of the same pseudogene into different orthogroups, thus sometimes artificially inflating the number of differences between genomes. In such cases, we merged the pseudogene entries in the Roary output before analyzing differences in gene content. Pseudogenized repeat elements (transposases and reverse transcriptases) were excluded from the manual curation due to their large number and the difficulty to infer orthology between genomes. Differences in gene content were also confirmed by visual inspection of sequencing depth in Artemis after reciprocally mapping the *w*AlbB-Uju and *w*AlbB-Hou (SRA accession no. SRR7623731) Illumina reads onto the two *w*AlbB genomes with Bowtie2 (Fig. S3). Using representative sequences, Tblastn searches were run to locate WO phage genes (WOVitA1 phage genome accession no. KX522565), including homologues of the Octomom and *cif* genes (WD0507-WD0514, WD0631, and WD0632 from *w*Mel genome accession no. AE017196.1). Prophage regions and their eukaryotic modules were then manually reannotated by following the most recent guidelines (75). Whole-genome and prophage region synteny were visualized using the R package genoPlotR (76). The maximum likelihood phylogenetic tree was inferred with RaxML v.7.7.6 (77) using a core gene alignment of several supergroup A and B *Wolbachia* genomes generated by Roary.

The SNP analysis was conducted using the Snippy pipeline v.4.6.0 (https://github.com/tseemann/snippy). Illumina reads were first down-sampled to reach a similar sequencing depth of the *Wolbachia* genome across samples (~100×). The remaining reads were then mapped onto the different *w*AlbB genomes, and SNPs were called with a threshold of 10 reads for minimum coverage, a 0.9 minimum proportion, >30 Phred quality scores for variant evidence (i.e., 99.9% accuracy), and a minimum mapping quality (MAPQ) of 20, except for reads with a MAPQ score of 0 to allow mapping to repetitive regions.

### Origin of *w*AlbB variants and mosquitoes used in phenotypic assays.

The *w*AlbB-Hou variant used in phenotypic comparisons originates from *Ae. aegypti* mosquitoes that were transinfected in 2005 (24) with the same *w*AlbB infection as the reference isolate found in the Aa23 *Ae. albopictus* cell line. *w*AlbB-Hou was then transinfected into an *Ae. aegypti* line with an Australian mitochondrial haplotype (44). wAlbB-Hou and wAlbB-Uju populations were backcrossed regularly to natively uninfected populations from Australia and Malaysia, respectively, to control for genetic background. Uninfected populations were created through antibiotic treatment, and the different combinations of nuclear background (Australian or Malaysian), mitochondrial haplotype (Australian or Malaysian) and *Wolbachia* infection status (wAlbB-Hou, wAlbB-Uju, or uninfected) were generated through reciprocal backcrosses as explained by Ross et al. (44). Two replicate populations of each combination were created and maintained separately. Both of these were included in *Wolbachia* density and quiescent egg viability measurements, while a single replicate population was tested for *Wolbachia* density under heat stress.

### *Wolbachia* detection and density.

Quantitative PCR (qPCR) assays were used to confirm the presence or absence of *Wolbachia* infection and measure relative density. Genomic DNA was extracted using 250 μL of 5% Chelex 100 resin (Bio-Rad laboratories, Hercules, CA) and 3 μL of proteinase K (20 mg/mL) (Roche Diagnostics Australia Pty., Ltd., Castle Hill, New South Wales, Australia). Tubes were incubated for 30 min at 65°C and then 10 min at 90°C. *Wolbachia* density was quantified by qPCR via the Roche LightCycler 480. Two primer sets were used to amplify markers specific to mosquitoes (forward primer mRpS6_F [5′-AGTTGAACGTATCGTTTCCCGCTAC-3′] and reverse primer mRpS6_R [5′-GAAGTGACGCAGCTTGTGGTCGTCC-3′]) and *w*AlbB (*w*AlbB_F [5′-CCTTACCTCCTGCACAACAA-3′] and *w*AlbB_R [5′-GGATTGTCCAGTGGCCTTA-3′]). For mosquitoes carrying the *w*Mel infection, *Wolbachia* density was determined using w1 primers (w1_F [5′-AAAATCTTTGTGAAGAGGTGATCTGC-3′] and w1_R [5′-GCACTGGGATGACAGGAAAAGG-3′]) (78). Relative *Wolbachia* densities were determined by subtracting the crossing point (Cp) value of the *Wolbachia*-specific marker from the Cp value of the mosquito-specific marker. Differences in Cp values were averaged across 2 to 3 consistent replicate runs and then transformed by 2^n^.

### Quiescent egg viability.

We measured quiescent egg viability in *Ae. aegypti* populations with different combinations of *w*AlbB infection type (*w*AlbB-Hou, *w*AlbB-Uju, or uninfected), mitochondrial haplotype (Au or My), and background (Au or My). Six cups filled with larval rearing water and lined with sandpaper strips were placed inside cages of blood fed females from each population. Eggs were collected 5 days after blood feeding, partially dried, then placed in a sealed chamber with an open container of saturated potassium chloride (KCl) solution to maintain a constant humidity of ~84%. When eggs were 1, 4, 7, 10, 13, and 16 weeks old, small sections of each sandpaper strip were removed and submerged in water with a few grains of yeast to hatch. Four to six replicate batches of eggs were hatched per replicate population at each time point, with 40 to 125 eggs per batch. Hatch proportions were determined by dividing the number of hatched eggs (with a clearly detached egg cap) by the total number of eggs per female.

### *Wolbachia* density following heat stress.

We measured *Wolbachia* density in adults after being exposed to cyclical heat stress during the egg stage. Eggs were collected from *Wolbachia*-infected populations (one replicate population each from *w*AlbB-Hou Au/Au, *w*AlbB-Hou Au/My, *w*AlbB-Uju My/Au, *w*AlbB-Uju My/My, and *w*Mel). Four days after collection, batches of 40 to 60 eggs were tipped into 0.2-mL PCR tubes (12 replicate tubes per population) and exposed to cyclical temperatures of 29 to 39°C for 7 days in Biometra TProfessional TRIO 48 thermocyclers (Biometra, Göttingen, Germany) according to Ross et al. (44). Eggs of the same age from each population were kept at 26°C. Eggs held at 29 to 39°C and 26°C were hatched synchronously, and larvae were reared at a controlled density (100 larvae per tray of 500 mL water). Pupae were sexed, and 15 males and 15 females per population and temperature treatment were stored in absolute ethanol within 24 h of emergence for *Wolbachia* density measurements (see “*Wolbachia* detection and density” above).

### Statistical analysis of density and phenotypic traits.

Experimental data were analyzed using SPSS Statistics version 24.0 for Windows (SPSS, Inc., Chicago, IL). Quiescent egg viability and *Wolbachia* density data were analyzed with general linear (mixed effect) models (GLMs). Replicate populations were pooled for analysis when effects of replicate population exceeded a *P* value of 0.1 in prior analyses. Data for each sex were analyzed separately. For *Wolbachia* density, untransformed data (i.e., differences in Cp between *Wolbachia* and mosquito markers, before 2^n^ transformation) were used for analyses. We ran additional GLMs on *Wolbachia* density in each nuclear background separately due to significant interactions between background and *w*AlbB variant. For comparisons of *Wolbachia* density at different temperatures, we included temperature treatment (26 or 26 to 39°C) as a factor. We were unable to perform direct comparisons between *w*Mel and *w*AlbB strains due to using different markers for each strain; we therefore excluded *w*Mel from the overall analysis. We ran separate GLMs for each *w*AlbB variant due to significant two-way interactions. For quiescent egg viability, hatch proportions differed substantially between *w*AlbB-infected and uninfected populations. We therefore ran separate GLMs for *w*AlbB-infected and uninfected populations, with egg storage duration included as an additional factor for this trait. Replicate population (nested within *Wolbachia* infection status) was included as a random factor due to significant effects of replicate population for this trait. Squared egg storage duration was also included as a factor in the GLM due to the nonlinear relationship between egg hatch proportion and storage duration in these populations.

### Multiplex PCR.

DNA was extracted from a pool of five female mosquitoes by crushing tissues in 200 μL of STE buffer (100 mM NaCl, 10 mM Tris-HCl, 1 mM EDTA [pH 8.0] Sigma-Aldrich). Each sample was then treated with 2 μL of proteinase K (20 mg/mL) at 65°C for 30 min, followed by a 10-min incubation step at 95°C. Tissue debris were removed by centrifugation for 2 min at 1,000 × *g*, and the supernatant was diluted 1/5 in water before PCR. Primer pairs specific for each *w*AlbB variant were designed to amplify a target gene present in one variant and absent in the other one. Primers were designed on an AAA family ATPase protein (DEJ70_04410; forward, 5′-ATGTCTGTTTCTGCGTCTTG-3′; reverse, 5′-ATCGTCTTTATCCAGCCCAG-3′; 303-bp product) for *w*AlbB-Hou and on the phage tail formation protein I for *w*AlbB-Uju (L3551_02370; forward, 5′-AGAAATACTGCGCTGGGTAA-3′; reverse, 5′-GGATTGCTACATCTAGGCGA-3′; 497-bp product). As a DNA extraction control, primers were also designed to amplify the mosquito 18S gene (forward, 5′-CCCAGCTGCTATTACCTTGA-3′; reverse, 5′-TAAGCAGAAGTCAACCACGA-3′; 752-bp product). The three primer pairs were pooled in a multiplex PCR using the Q5 high-fidelity DNA polymerase (New England Biolabs) in a 25-μL final volume as follows: 5 μL of buffer, 0.5 of 10 mM deoxynucleoside triphosphates (dNTPs), 1.25 μL of each 10 μM primer, 0.25 μL of DNA polymerase, 9.75 μL of water, and 2 μL of DNA template. The PCR cycle used was 98°C for 30s, 35 cycles of 10 s of denaturation at 98°C, 30s of annealing at 64°C, and a 1-min extension at 72°C, followed by a 2-min final extension at 72°C. PCR products were run on 1% agarose gel electrophoresis.

### Data availability.

The *w*AlbB-Uju draft genome and raw sequencing data have been deposited in the NCBI GenBank database under the BioProject accession no. PRJNA800254 (assembly CP102671; Illumina reads SRR17831854 to SRR17832810; Oxford Nanopore reads SRR21023725).
