# Supplementary material for: Genomic and Phenotypic Comparisons Reveal Distinct Variants of Wolbachia Strain wAlbB
Source: Appl Environ Microbiol. 2022 Nov 1;88(22):e01412-22. doi: 10.1128/aem.01412-22 (PMC9680635; doi:10.1128/aem.01412-22)
Supplement: Supplemental file 1 — Fig. S1 to S3 and Tables S3 to S5. Download aem.01412-22-s0001.pdf, PDF file, 1.0 MB [file aem.01412-22-s0001.pdf]

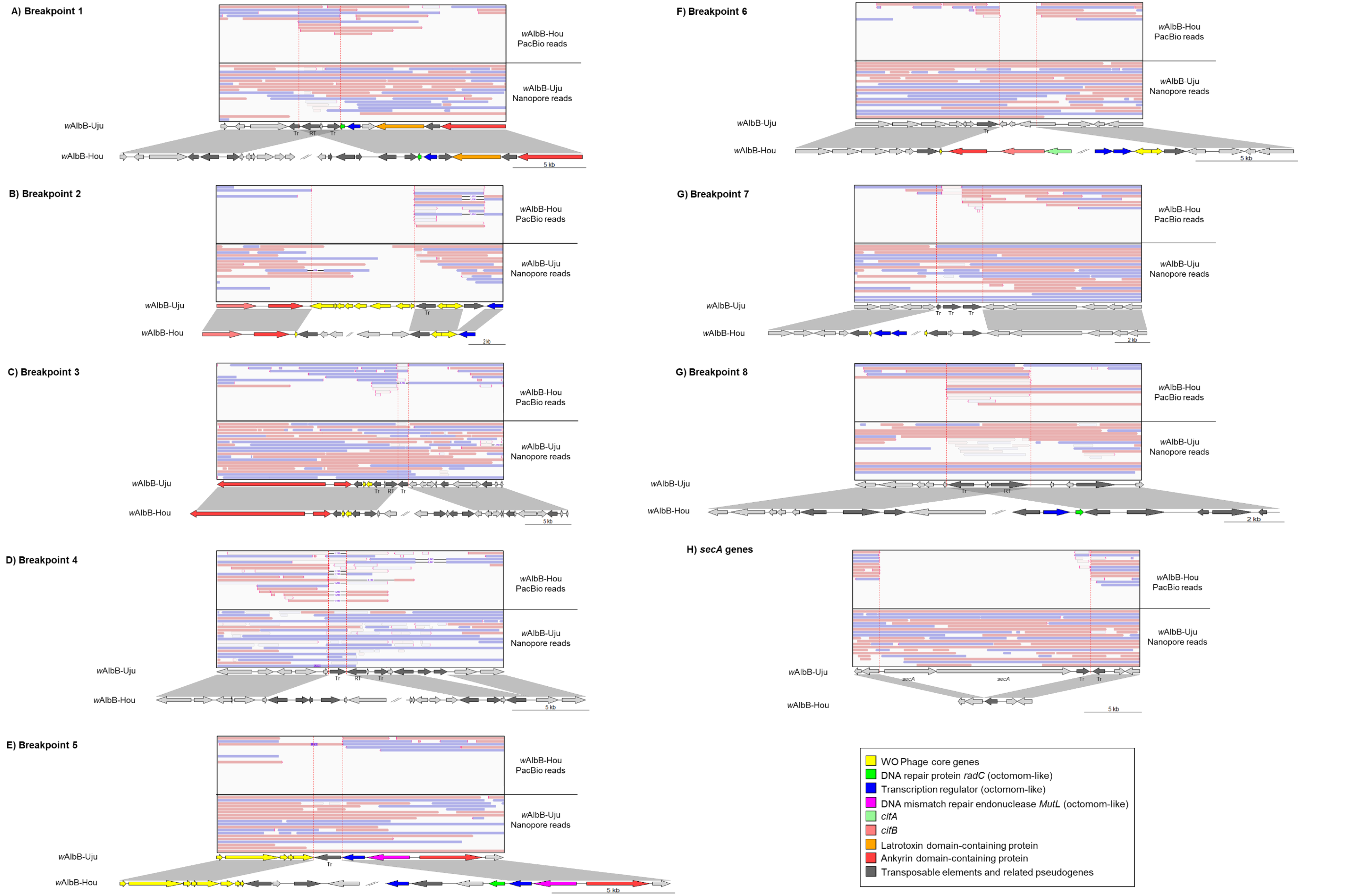

Figure S1. Analysis of chromosomal breakpoints between *w*AlbB-Uju and *w*AlbB-Hou. Alignment boxes display long reads from both variants (SRA accessions: SRR21023725 and SRR7784287) aligned to the *w*AlbB-Uju genome at chromosomal breakpoints (A-G) and a large indel comprising horizontally-transferred *SecA* genes (H). Dashed red lines indicate chromosomal breakpoints. Tr: transposase ; RT: reverse transcriptase.

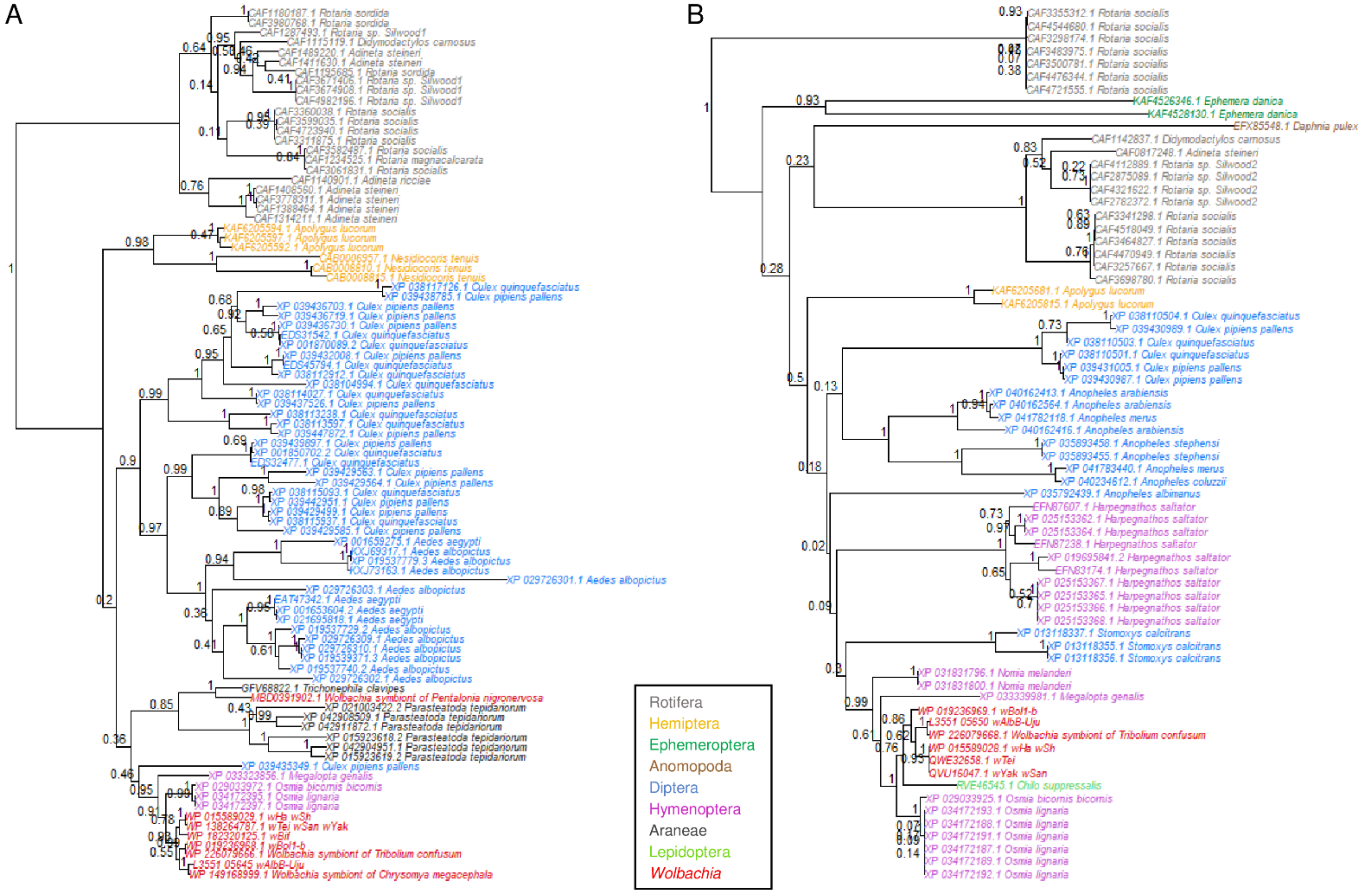

Figure S2. Maximum likelihood phylogenies of horizontally-transferred translocase subunit *SecA* genes. (A) L3551_05645 and (B) L3551_05650 amino acids were aligned with their homologues and conserved sites (Gblocks) were used to build the phylogenies with PhyML. Node labels are bootstrap supports calculated from 100 replicates.

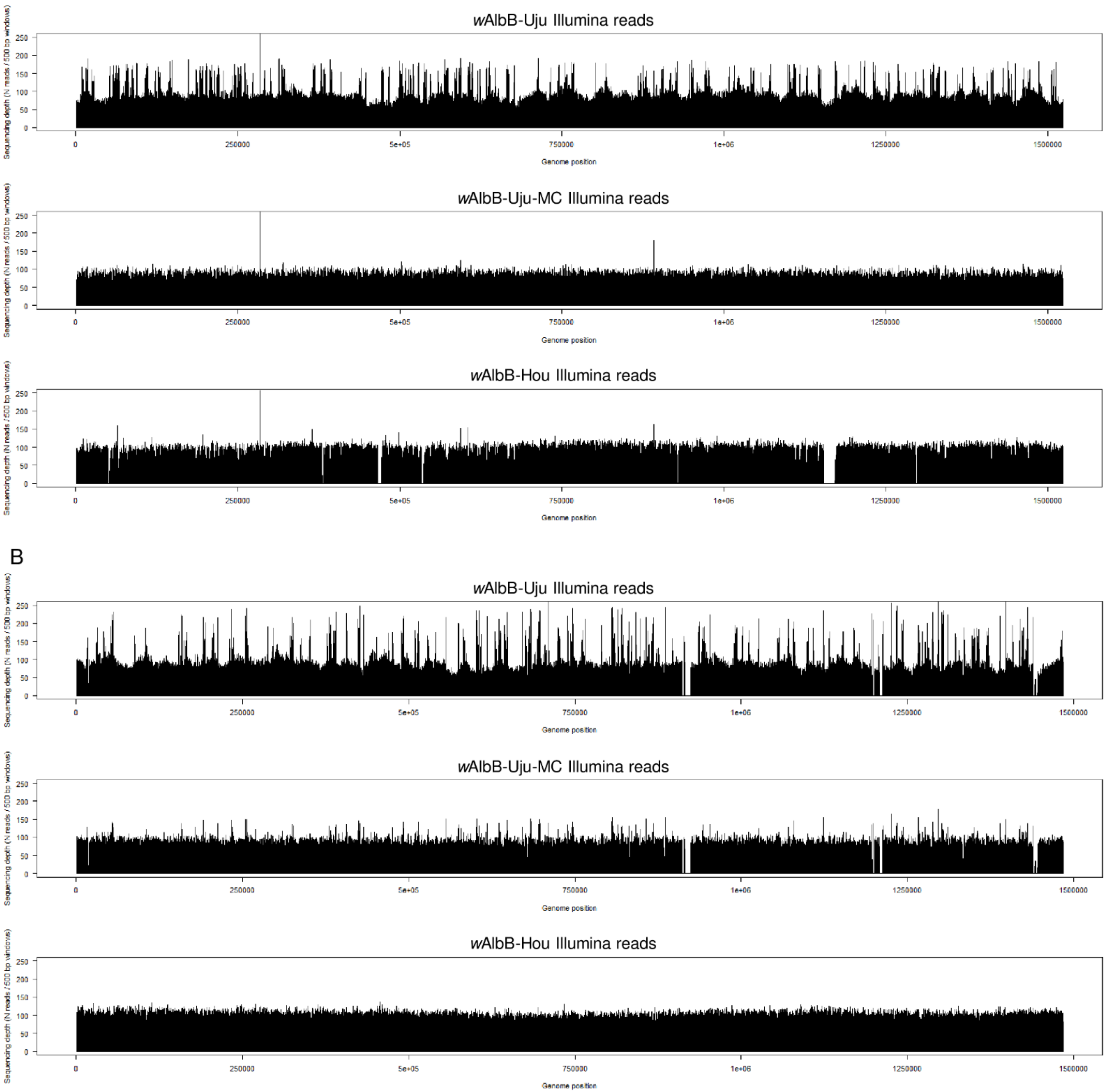

Figure S3. Sequencing depth plots. Mean sequencing depth per 500 bp window of Illumina reads mapped onto (A) *w*AlbB-Uju and (B) *w*AlbB-Hou genomes.

Trait	Factor	Type III Sum of Squares	df	Mean Square	F	P
Female *Wolbachia* density	*w*AlbB variant	2.863	1	2.863	11.068	**0.001**
	Nuclear background	0.637	1	0.637	2.464	0.119
	Error	39.57	153	0.259		
Male *Wolbachia* density	*w*AlbB variant	4.764	1	4.764	24.111	**< 0.0001**
	Nuclear background	0.216	1	0.216	1.093	0.298
	*w*AlbB variant by nuclear background	1.688	1	1.688	8.541	**0.004**
	Error	29.442	149	0.198		

Table S3. Statistical analysis of differences in density between *w*AlbB variants.

Sex	Factor	Type III Sum of Squares	df	Mean Square	F	P
Female	*w*AlbB variant	64.79	1	64.79	71.089	**< 0.0001**
	Nuclear background	4.738	1	4.738	5.199	**0.024**
	Temperature	41.206	1	41.206	45.211	**< 0.0001**
	*w*AlbB variant by Nuclear background	13.486	1	13.486	14.797	**< 0.0001**
	*w*AlbB variant by Temperature	28.611	1	28.611	31.393	**< 0.0001**
	Error	103.899	114	0.911		
Male	*w*AlbB variant	55.897	1	55.897	95.736	**< 0.0001**
	Nuclear background	1.162	1	1.162	1.991	0.161
	Temperature	30.633	1	30.633	52.467	**< 0.0001**
	*w*AlbB variant by Nuclear background	3.478	1	3.478	5.957	**0.016**
	*w*AlbB variant by Temperature	28.237	1	28.237	48.362	**< 0.0001**
	Error	66.56	114	0.584		

Table S4. Statistical analysis of *w*AlbB variant densities under heat stress.

Population comparison	Factor		Type III Sum of Squares	df	Mean Square	F	P
*w*AlbB-infected populations	Egg storage duration^2	Hypothesis	14.518	1	14.518	78.446	**< 0.0001**
		Error	41.27	223	0.185		
	Egg storage duration	Hypothesis	2.067	1	2.067	11.17	**0.001**
		Error	41.27	223	0.185		
	*w*AlbB variant	Hypothesis	7.815	1	7.815	12.236	0.072
		Error	1.284	2.01	0.639		
	Nuclear background	Hypothesis	1.754	1	1.754	9.475	**0.002**
		Error	41.27	223	0.185		
	Replicate population within *w*AlbB variant	Hypothesis	1.285	2	0.643	3.472	**0.033**
		Error	41.27	223	0.185		
	*w*AlbB variant by Nuclear background	Hypothesis	0.694	1	0.694	3.75	0.054
		Error	41.27	223	0.185		

Table S5. Statistical of quiescent egg viability.
